# Multimodal Anterior Segment Imaging of Severe Mixed Exposure-Related Neurotrophic Keratopathy with Marked Corneal Thinning in Lamellar Ichthyosis

**DOI:** 10.3390/diagnostics16081209

**Published:** 2026-04-17

**Authors:** Wojciech Luboń, Małgorzata Luboń, Mariola Dorecka

**Affiliations:** 1 Department of Ophthalmology, Faculty of Medical Sciences, Medical University of Silesia, 40-514 Katowice, Poland; mdorecka@sum.edu.pl; 2 Department of Ophthalmology, Professor K. Gibiński University Clinical Center, Medical University of Silesia, 40-514 Katowice, Poland; kozikowskamalg@gmail.com

**Keywords:** lamellar ichthyosis, neurotrophic keratopathy, corneal ulcer, corneal thinning, anterior segment OCT, slit-lamp imaging, exposure keratopathy, ectropion

## Abstract

Lamellar ichthyosis is a rare congenital disorder of keratinization frequently associated with ocular complications, most commonly cicatricial ectropion and exposure keratopathy. We present a case of severe mixed exposure-related and neurotrophic keratopathy with marked corneal thinning in a 61-year-old man with genetically confirmed lamellar ichthyosis. At presentation, the best-corrected visual acuity (BCVA) in the right eye was limited to hand motion (logMAR 2.3). Slit-lamp examination revealed a large central to inferocentral corneal ulcer measuring approximately 3 × 4 mm with severe stromal thinning in the setting of marked lower eyelid ectropion, incomplete eyelid closure, and chronic ocular surface exposure, while anterior segment optical coherence tomography (AS-OCT) demonstrated a minimal corneal thickness of approximately 165 µm. Microbiological swabs obtained from the conjunctival sac were negative, and no purulent discharge, hypopyon, or anterior chamber inflammatory reaction was present, making active infectious keratitis unlikely. Corneal sensitivity measured with Cochet–Bonnet esthesiometry at presentation, centrally and in all four peripheral quadrants of both eyes, was markedly reduced, more severely in the affected right eye, supporting the presence of a severe neurotrophic component contributing to impaired corneal healing. Intensive conservative therapy including preservative-free lubricants, dexpanthenol gel, autologous serum eye drops, topical insulin, prophylactic antibiotics, and systemic doxycycline was initiated. Serial AS-OCT imaging demonstrated progressive structural recovery, with corneal thickness increasing to 438 µm after one month of treatment and complete corneal epithelialization. The BCVA improved to 0.2 Snellen (0.7 logMAR). This case highlights the diagnostic value of multimodal anterior segment imaging in monitoring severe mixed keratopathy with advanced corneal thinning and demonstrates that intensive conservative therapy may stabilize the ocular surface and prevent corneal perforation in patients with lamellar ichthyosis.

**Figure 1:**
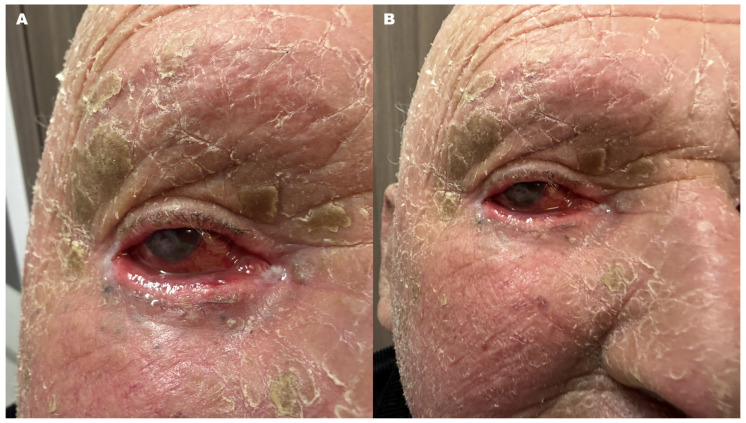
Clinical photographs of a 61-year-old patient with genetically confirmed lamellar ichthyosis demonstrating characteristic dermatological and ocular manifestations of the disease. Facial images (**A**,**B**) show diffuse hyperkeratosis with thick adherent scales and generalized xerosis involving the skin of the face and scalp. Bilateral lower eyelid ectropion is present, more pronounced in the right eye, resulting in incomplete eyelid closure and chronic ocular surface exposure. The right eye demonstrates marked conjunctival hyperemia and ocular surface inflammation. A central corneal ulcer with surrounding stromal opacity is visible, consistent with severe mixed exposure-related and neurotrophic keratopathy with a prominent exposure-related component. Chronic eyelid malposition in lamellar ichthyosis frequently leads to persistent ocular surface exposure, which may result in epithelial breakdown, secondary corneal ulceration, and progressive stromal damage. In advanced cases, exposure keratopathy and neurotrophic keratopathy may overlap clinically, as chronic surface desiccation promotes epithelial failure, while impaired corneal innervation further limits corneal repair, increasing the risk of stromal melting and perforation if untreated [1,2,3].

**Figure 2:**
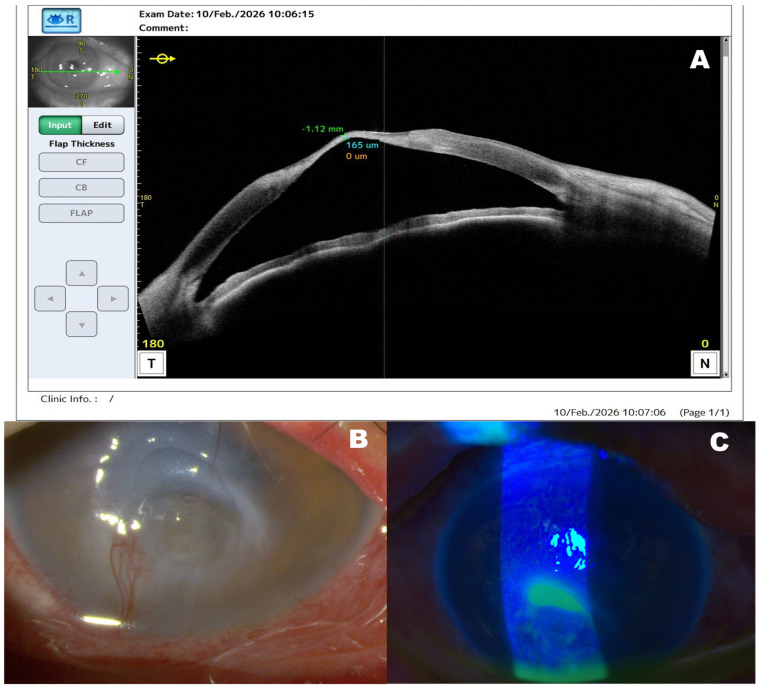
Anterior segment imaging demonstrating severe corneal ulceration and marked corneal thinning associated with mixed exposure-related and neurotrophic keratopathy in lamellar ichthyosis. Anterior segment optical coherence tomography (AS-OCT; CASIA2, Tomey Corporation, Nagoya, Japan) scan (**A**) obtained at admission demonstrates pronounced stromal thinning in the central cornea, with a minimal corneal thickness of approximately 165 µm, indicating a high risk of corneal perforation. Slit-lamp photograph (**B**) of the right eye shows a large central corneal ulcer with surrounding stromal opacity and significant corneal thinning. Prominent superficial corneal neovascularization is visible together with irregular epithelialization in the superior corneal region adjacent to the central ulcer. Slit-lamp image (**C**) obtained under cobalt-blue illumination following fluorescein staining demonstrates intense fluorescein pooling within the ulcer bed, confirming the presence of a deep corneal epithelial defect with stromal involvement. In this patient, marked bilateral cicatricial ectropion, incomplete eyelid closure, and inferior ocular surface dryness indicated advanced exposure-related ocular surface disease, while Cochet–Bonnet esthesiometry performed at presentation in both eyes demonstrated markedly reduced corneal sensitivity, more severe in the right eye, supporting a coexisting neurotrophic component. Before referral, the patient had been treated for approximately 3 months with lubricants and topical aminoglycoside antibiotics without clinical resolution. Conjunctival sac microbiological swabs were negative, and no purulent discharge, hypopyon, or anterior chamber inflammatory reaction was present, making active bacterial keratitis unlikely. Due to the high risk of perforation, intensive conservative therapy was initiated, including preservative-free lubricants, dexpanthenol gel, topical antibiotic prophylaxis, autologous serum eye drops, topical insulin, and systemic doxycycline to inhibit stromal collagen degradation. Persistent ocular surface exposure in lamellar ichthyosis may lead to progressive corneal ulceration and, in advanced cases, stromal melting or perforation, particularly when accompanied by impaired corneal innervation [4,5].

**Figure 3:**
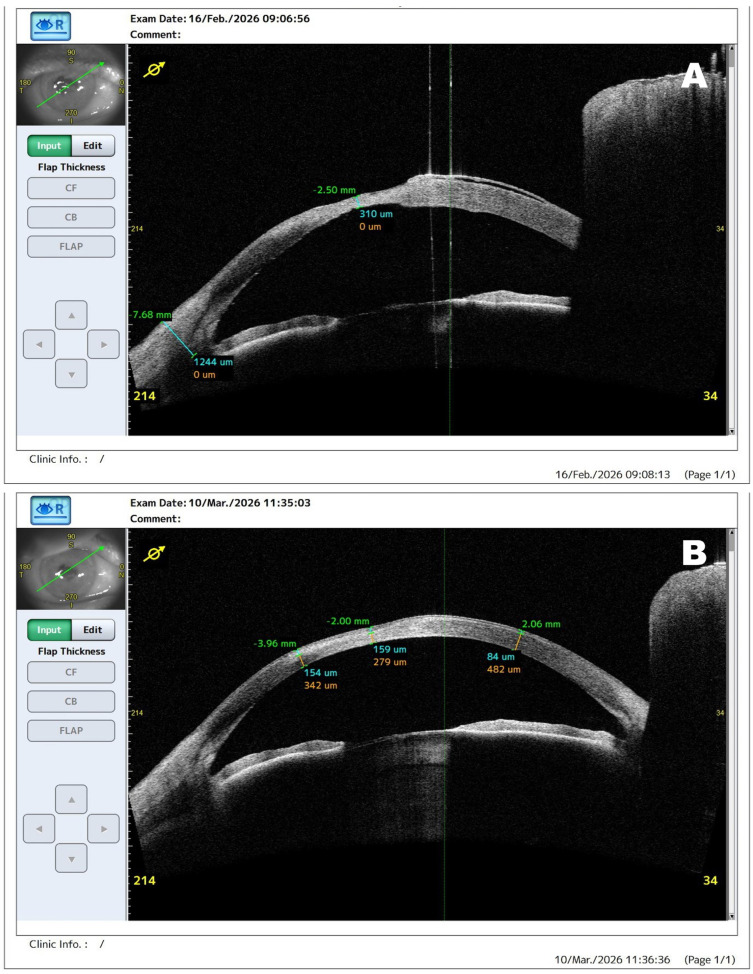
Serial AS-OCT images of the right eye demonstrating progressive restoration of corneal thickness after intensive conservative therapy. AS-OCT (**A**), obtained six days after treatment initiation, shows an increase in corneal thickness at the previously thinnest location to approximately 310 µm. Follow-up imaging (**B**) one month later demonstrates further stromal thickening with minimal corneal thickness of approximately 438 µm, indicating stabilization of the corneal structure. Serial anterior segment imaging may play an important role in monitoring corneal healing and guiding therapeutic decisions in severe ocular surface disease associated with lamellar ichthyosis [6,7].

**Figure 4:**
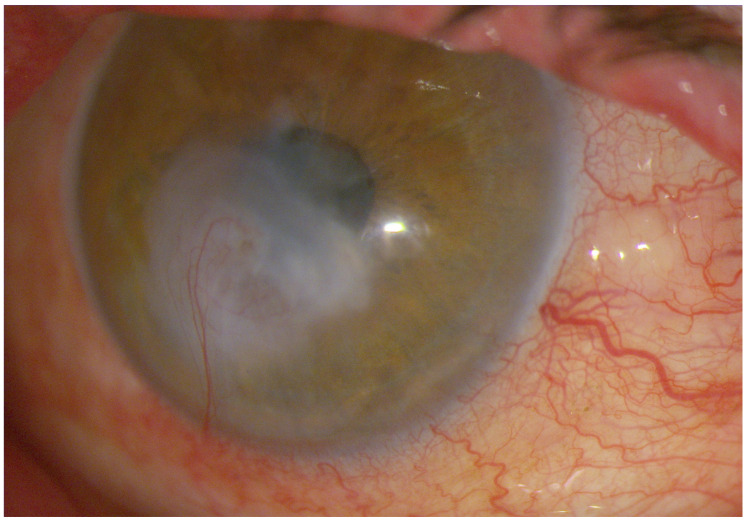
High-magnification slit-lamp photograph of the right eye obtained during follow-up showing the final stage of corneal healing after intensive conservative therapy. A central corneal scar covered by regenerated epithelium is visible, with no signs of a persistent epithelial defect. Peripheral corneal neovascularization extending toward the area of previous ulceration is present, representing a chronic reparative response. During follow-up, BCVA improved from hand motion at presentation to 0.7 logMAR. Surgical correction of the lower eyelid ectropion has been planned to address the persistent exposure-related component of the ocular surface disease and to reduce the risk of recurrent surface decompensation. Management of severe mixed exposure-related and neurotrophic keratopathy requires both support of corneal healing and correction of the underlying ocular surface imbalance. Depending on disease severity, treatment options may include intensive lubrication, autologous serum, topical insulin, anti-collagenolytic therapy, therapeutic amniotic membrane transplantation (AMT), and surgical correction of eyelid malposition. Maintenance therapy included topical insulin, autologous serum eye drops, preservative-free artificial tears, topical dexamethasone, and systemic doxycycline [8,9].

## Data Availability

The original contributions presented in this study are included in the article. Further inquiries can be directed to the corresponding author.

## Abbreviations

The following abbreviations are used in this manuscript:

AS-OCT	Anterior Segment Optical Coherence Tomography
AMT	Amniotic Membrane Transplantation
BCVA	Best-Corrected Visual Acuity

## References

[1] Al-Amry M.A. (2016). Ocular manifestation of Ichthyosis. Saudi J. Ophthalmol..

[2] Malhotra R., Hernández-Martın A., Oji V. (2018). Ocular manifestations, complications and management of congenital ichthyoses: A new look. Br. J. Ophthalmol..

[3] Bhedasgaonkar S.S., Nadkarni S.U. (2020). Corneal ulcer secondary to ectropion in lamellar Ichthyosis: A rare congenital disorder. Saudi J. Ophthalmol..

[4] Zdebik A., Zdebik N., Fischer M. (2021). Ocular manifestations of skin diseases with pathological keratinization abnormalities. Postepy Dermatol. Alergol..

[5] Khadamy J. (2025). Ocular Manifestations Leading to the Diagnosis of Ichthyosis: A Case Report. Cureus.

[6] Macriz-Romero N., Vera-Duarte G.R., Guerrero-Becerril J., Chacón-Camacho O.F., Astiazarán M.C., Zenteno J.C., Graue-Hernandez E.O. (2023). Ophthalmic findings in patients with autosomal recessive lamellar ichthyosis due to TGM1 mutations in an isolated population. Int. Ophthalmol..

[7] Micińska A., Nowińska A., Teper S., Kokot-Lesik J., Wylęgała E. (2023). Advanced Anterior Eye Segment Imaging for Ichthyosis. J. Clin. Med..

[8] Moustaine M.O., Frarchi M., Haloui M., Chabbab F.Z. (2022). Severe Bilateral Ectropion in Lamellar Ichthyosis: A Case Report. Am. J. Case Rep..

[9] Rossetto J.D., Gracitelli C.P.B., Osaki T.H., Osaki M.H. (2019). Diseases, conditions, and drugs associated with cicatricial ectropion. Arq. Bras. Oftalmol..

